# Persistent Disparities in Immunization Rates for the Seven-Vaccine Series Among Infants 19–35 Months in the United States

**DOI:** 10.1089/heq.2020.0127

**Published:** 2021-03-16

**Authors:** Ansh A. Kulkarni, Raj P. Desai, Héctor E. Alcalá, Rajesh Balkrishnan

**Affiliations:** ^1^ Department of Public Health Sciences, University of Virginia School of Medicine, Charlottesville, Virginia, USA.; ^2^ Department of Family, Population and Preventive Medicine, Stony Brook University, Stony Brook, New York, USA.

**Keywords:** childhood, disparities, epidemiology, United States, vaccination

## Abstract

**Objective:** The seven-vaccine series protects infants from several preventable diseases, yet disparities in its use remain in the United States.

**Methods:** We assessed the seven-vaccine immunization rate and its predictors in infants 19–35 months using the National Immunization Survey from 2009 to 2018.

**Results:** The seven-vaccine series rate was 72.8%, well short of the healthy people 2020 target of 90%. African American infants, infants born to mothers with less than high school education, and infants in families with an income below poverty were less likely to get the complete series.

**Conclusion:** Disparities still exist in protecting infants from preventable diseases in the United States.

## Introduction

The combined seven-vaccine series (4:3:1:3/4:3:1:4) includes ≥4 doses of DTaP, ≥3 doses of poliovirus vaccine, ≥1 dose of measles-containing vaccine, the full series of Hib (≥3 or ≥4 doses, depending on product type), ≥3 doses of HepB, ≥1 dose of varicella vaccine, and ≥4 doses of PCV. These seven-vaccine series provide immunization against diphtheria, pertussis, tetanus, poliovirus, measles, mumps, rubella, hepatitis b, hemophilus influenza b, chicken pox, and pneumococcal infections. Failure to become vaccinated to any or all of the vaccines may lead to increased risk of infection, illness, death, and a decrease in the overall immunity of the total population. The office of disease prevention and health promotion (a part of the U.S. Department of Health and Human Service) has set a target of 90% completion for each of the seven vaccines through its Healthy People 2020 initiative.^1^ The ongoing global coronavirus disease 2019 (COVID-19) pandemic has brought vaccination to the front and center of health policy discussion, especially in tackling infectious diseases. Despite national attention toward the coronavirus vaccine, barriers to receiving health care as a result of the ongoing pandemic have led to a decrease in immunization against preventable diseases.^2^ The objective of this study was to assess the seven-vaccine immunization rate in infants 19–35 months over a decade spanning 2009 to 2018 and to understand factors that affect immunization, and to provide the most recent benchmark for the seven-vaccine series immunization rate during the “pre” COVID-19 pandemic time period.

## Methods

The National Immunization Survey (NIS) is conducted annually by the Centers for Disease Control and Prevention (CDC) to obtain national, state, and selected urban area estimates of vaccination coverage for U.S. children aged 19 to 35 months at the time of household interview. The NIS is a random-digit-dial survey of households followed by a mail survey to all of the children's immunization providers to verify the vaccination information.^3^

Demographic characteristics, including maternal age, education, marital status, child's age, race/ethnicity, birth order, and poverty status, were reported by respondents. Up-to-date vaccination coverage was defined as receipt of the combined seven-vaccine series for children aged 19–35 months at the time of the household interview. This study is considered IRB exempt as it uses publicly available deidentified data.

Chi-square analyses were performed to test for associations between child and parental variables and vaccination coverage. Weighted logistic regression analyses tested for associations with the vaccination coverage status while controlling for demographic variables; adjusted odds ratios (ORs) and 95% confidence intervals (CIs) are reported. A two-sided significance level of 0.05 was adopted for all statistical tests. All analyses were conducted using SAS, release 9.4 (SAS Institute, Cary, NC).

## Results

Although the combined seven-vaccine series rate has increased by ∼30% over the 10-year period from 2009 to 2018, only 72.8% of infants aged 19–35 months had received all of the recommended seven-vaccine series in 2018, far from the healthy people 2020 goal of 90% (Table 1).^1^ As compared with non-Hispanic whites, non-Hispanic blacks were less likely to receive the seven-vaccine series (OR: 0.92; 95% CI: 0.86–0.98), whereas Hispanics were more likely to receive the seven-vaccine series (OR: 1.22; 95% CI: 1.15–1.30) (Table 2). Infants from families with income below the poverty threshold were ∼30% less likely to receive the seven-vaccine series as compared with those with income >75k USD per year (OR: 0.69; 95% CI: 0.65–0.75). A comparison of the analysis from the NIS data from years 2009 to 2018 showed that the impact of income on the seven-vaccine series rate has increased (2009: 9% less likely to 2018: 37% less likely to complete the seven-vaccine series as compared with families with income >$75k). Infants with mothers with less than high school education were almost 27% less likely to receive the seven-vaccine series as compared with infants with mothers who had a college degree (OR: 0.73; 95% CI: 0.67–0.79).

**Table 1.** Characteristics of Children Aged 19–35 Months by Seven-Vaccine Series Completion Status—National Immunization Survey—Child, United States, 2009–2018 (*n*=159,331)

Characteristics	Overall		Yes		No		*p*
	Sample size^a^	Weighted % (95% CI)	Sample size^a^	Weighted % (95% CI)	Sample size^a^	Weighted % (95% CI)	
Total	159,331	100.0 (—)	108,291	66.3 (65.9–66.8)	51,040	33.7 (33.2–34.1)	<0.0001
Survey year							<0.0001
2009	17,053	10.7 (10.5–11.0)	7432	7.2 (6.9–7.4)	9621	17.8 (17.2–18.3)	
2010	16,798	10.5 (10.3–10.7)	9715	9.0 (8.7–9.2)	7083	13.5 (13.0–14.0)	
2011	19,144	10.2 (10.0–10.5)	13,375	10.6 (10.2–10.9)	5769	9.5 (9.1–10.0)	
2012	16,687	9.9 (9.6–10.2)	11,836	10.2 (9.9–10.5)	4851	9.3 (8.8–9.8)	
2013	13,611	9.8 (9.5–10.1)	9813	10.4 (10.0–10.7)	3798	8.6 (8.1–9.1)	
2014	14,893	9.7 (9.4–10.0)	10,933	10.5 (10.1–10.9)	3960	8.2 (7.7–8.7)	
2015	15,167	9.7 (9.4–10.0)	11,322	10.6 (10.2–10.9)	3845	8.0 (7.6–8.4)	
2016	14,988	9.8 (9.5–10.1)	11,000	10.5 (10.1–10.8)	3988	8.5 (8.0–9.0)	
2017	15,333	9.8 (9.5–10.1)	11,174	10.5 (10.1–10.8)	4159	8.6 (8.2–9.1)	
2018	15,657	9.8 (9.5–10.1)	11,691	10.8 (10.4–11.1)	3966	7.9 (7.4–8.4)	
Age (years)							<0.0001
19–23 months	45,935	30.0 (29.5–30.4)	28,886	27.8 (27.2–28.3)	17,049	34.4 (33.6–35.2)	
24–29 months	50,487	33.9 (33.5–34.4)	34,646	34.5 (34.0–35.1)	15,841	32.7 (31.9–33.5)	
30–35 months	62,909	36.1 (35.6–36.5)	44,759	37.7 (37.1–38.3)	18,150	32.9 (32.1–33.7)	
Race/ethnicity							<0.0001
Hispanic	30,823	27.2 (26.7–27.7)	20,878	27.1 (26.5–27.7)	9945	27.3 (26.4–28.1)	
Non-Hispanic white	94,627	47.8 (47.3–48.3)	64,889	48.4 (47.8–49.0)	29,738	46.6 (45.8–47.5)	
Non-Hispanic black	14,264	13.0 (12.6–13.3)	9060	12.1 (11.7–12.5)	5204	14.7 (14.1–15.3)	
Non-Hispanic other	19,617	12.0 (11.7–12.4)	13,464	12.4 (12.0–12.8)	6153	11.4 (10.9–11.9)	
Child was firstborn							<0.0001
No	95,677	59.1 (58.6–59.6)	63,339	57.3 (56.7–57.8)	32,338	62.7 (61.9–63.5)	
Yes	63,654	40.9 (40.4–41.4)	44,952	42.7 (42.2–43.3)	18,702	37.3 (36.5–38.1)	
Poverty status (based on FPL)	60,795	28.5 (28.1–28.9)	44,714	31.6 (31.1–32.2)	16,081	22.2 (21.6–22.9)	<0.0001
Above FPL (>$75,000)	55,881	33.8 (33.3–34.2)	36,709	33.1 (32.6–33.7)	19,172	35.1 (34.3–35.9)	
Above FPL (up to $75,000)	37,106	31.9 (31.5–32.4)	23,252	29.5 (29.0–30.1)	13,854	36.7 (35.8–37.5)	
Below FPL	5549	5.8 (5.5–6.1)	3616	5.7 (5.4–6.0)	1933	6.0 (5.5–6.4)	
Unknown							
Marital status of mother							<0.0001
Married	116,631	63.6 (63.1–64.1)	80,832	65.2 (64.6–65.8)	35,799	60.4 (59.6–61.2)	
Never married/widowed/divorced/separated/deceased	42,700	36.4 (35.9–36.9)	27,459	34.8 (34.2–35.4)	15,241	39.6 (38.8–40.4)	
Education level of mother							<0.0001
<12 years	17,074	17.6 (17.2–18.0)	10,368	15.9 (15.4–16.4)	6706	21.1 (20.3–21.8)	
12 years	28,108	26.7 (26.2–27.2)	17,893	25.4 (24.8–25.9)	10,215	29.3 (28.5–30.1)	
>12 years, noncollege graduate	40,492	21.8 (21.4–22.2)	26,703	21.7 (21.3–22.2)	13,789	22.0 (21.3–22.6)	
College graduate	73,657	33.8 (33.4–34.3)	53,327	37.0 (36.5–37.6)	20,330	27.6 (26.9–28.3)	
Mother's age (years)							<0.0001
≤29	54,643	41.2 (40.7–41.7)	34,987	38.7 (38.1–39.3)	19,656	46.0 (45.2–46.8)	
≥30	104,688	58.8 (58.3–59.3)	73,304	61.3 (60.7–61.9)	31,384	54.0 (53.2–54.8)	
Census region							0.1490
Northeast	29,450	16.0 (15.8–16.1)	20,519	16.1 (15.8–16.4)	8931	15.7 (15.2–16.1)	
Midwest	34,369	20.8 (20.6–21.1)	23,654	20.9 (20.6–21.2)	10,715	20.8 (20.3–21.3)	
South	59,851	38.5 (38.2–38.8)	40,999	38.7 (38.2–39.1)	18,852	38.2 (37.5–38.9)	
West	35,661	24.7 (24.3–25.1)	23,119	24.4 (23.8–24.9)	12,542	25.3 (24.5–26.2)	

^a^ Unweighted sample size.

CI, confidence interval; FPL, federal poverty level.

**Table 2.** Factors Associated with Not Receiving Seven-Vaccine Series —National Immunization Survey—Child, United States, 2009–2018 (*n*=159,331)

Characteristics	Odds ratio	Standard error	95% CI
Survey year			
2009	Reference	Reference	Reference
2010	1.65	0.0406	1.53–1.79
2011	2.94	0.0421	2.71–3.20
2012	2.89	0.0441	2.65–3.15
2013	3.14	0.0470	2.86–3.44
2014	3.34	0.0471	3.04–3.66
2015	3.37	0.0453	3.08–3.68
2016	3.07	0.0479	2.80–3.38
2017	3.03	0.0468	2.76–3.32
2018	3.34	0.0502	3.03–3.68
Age (years)			
19–23 months	0.70	0.0263	0.66–0.73
24–29 months	0.92	0.0262	0.88–0.97
30–35 months	Reference	Reference	Reference
Race/ethnicity			
Hispanic	1.22	0.0314	1.15–1.30
Non-Hispanic white	Reference	Reference	Reference
Non-Hispanic black	0.92	0.0348	0.86–0.98
Non-Hispanic other	1.04	0.0336	0.97–1.11
Child was firstborn			
No	Reference	Reference	Reference
Yes	1.35	0.0229	1.29–1.41
Poverty status			
Above FPL (>$75,000)	Reference	Reference	Reference
Above FPL (up to $75,000)	0.75	0.0294	0.71–0.80
Below FPL	0.70	0.0378	0.65–0.75
Unknown	0.77	0.0568	0.69–0.86
Marital status of mother			
Married	Reference	Reference	Reference
Never married/widowed/divorced/separated/deceased	0.99	0.0284	0.94–1.05
Education level of mother			
<12 years	0.73	0.0410	0.67–0.79
12 years	0.85	0.0341	0.79–0.91
>12 years, noncollege graduate	0.89	0.0301	0.84–0.94
College graduate	Reference	Reference	Reference
Mother's age (years)			
≤29	0.82	0.0252	0.78–0.86
≥30	Reference	Reference	Reference
Census region			
Northeast	0.92	0.0260	0.88–0.97
Midwest	0.97	0.0239	0.92–1.01
South	Reference	Reference	Reference
West	0.88	0.0345	0.83–0.94

## Discussion

The low seven-vaccine series rates in low-income (below poverty) families are disheartening, especially with federal programs such as Vaccine for Children (VFC). VFC provides free vaccines for uninsured, underinsured, and Medicaid eligible children. Although vaccines themselves are free through the VFC program, the physician can potentially charge a fee to administer vaccines or for the office visit or other nonvaccine services.^4^ Free vaccination coupled with no additional fees, linked with potential programs that are frequently accessed by low-income families, could be a potential solution to increase the seven-vaccine series rates and should be considered. Such programs in the past have been implemented with some success.^5^ Pharmacists too are well positioned to provide vaccinations. Ninety percent of Americans stay within 5 miles of a community pharmacy.^6^ In the wake of the COVID-19 pandemic and ensuing complications, a drop in childhood vaccination rates^7^ prompted health and human services to allow pharmacists in all 50 states to immunize children >3 years of age.^8^ There is recognition that immunization of children <3 years may pose complications in a pharmacy setting.^6^ However, with appropriate continuing education credits, training of current pharmacists, and pharmacy schools providing training to future pharmacist students in infant/children vaccinations, these barriers can be potentially overcome.

A previous study has assessed the link between maternal education and child immunization using NIS data from almost two decades ago (1995–2003).^9^ Their study estimated that mothers with less than high school education were 7.8% less likely to be up to date with their six-vaccine series. Our study assessed that mothers with less than high school education were almost 27% less likely to receive the seven-vaccine series as compared with mothers with college education. The CDC, through its task force on community prevention services, identifies three areas to increase vaccination rates: increasing demand for vaccination within the community, increasing access to vaccination services, and provider-based intervention.^10^ The CDC recommends that health care professionals use well visits as a time to discuss and have dialogue on vaccination with the parents.^11^ However, disparities also exist in seeking and getting access to primary care.^12^ Studies have also suggested that language barriers and lack of knowledge on immunization contribute to children not getting immunized.^10^ Programs that address all these shortcomings should target mothers with an education level of high school or lower.

Our study also showed that African American infants were less likely to receive the seven-vaccine series. Such disparities, in this age, especially in protecting infants from potentially preventable diseases, are unacceptable. Previous research has highlighted several reasons for this disparity among the African American population, including lack of access to preventive health care, lack of trust in the health care system, and lack of understanding of the risks and benefits of vaccinations.^13^ A study by Wood, et al., showed the impact of case manager-based education on vaccination rates of inner city African American children in Los Angeles.^14^ Although this study was successful in increasing the immunization rates, the intervention itself was deemed not cost effective. More cost-effective methods need to be put in place to increase immunization rates among the African American community. Although the seven-vaccine series immunization rates in infants have increased in the United States over the past 10 years, disparities still exist in protecting infants from preventable diseases. These disparities negate the success of the increased vaccination rate.

## Abbreviations Used

CDC: Centers for Disease Control and Prevention
CI: confidence interval
COVID-19: coronavirus disease 2019
NIS: National Immunization Survey
OR: odds ratio
VFC: Vaccine for Children
